# Towards the clinical implementation of pharmacogenetics in bipolar disorder

**DOI:** 10.1186/1741-7015-12-90

**Published:** 2014-05-30

**Authors:** Naji C Salloum, Michael J McCarthy, Susan G Leckband, John R Kelsoe

**Affiliations:** 1 Department of Psychiatry (0603), University of California San Diego, La Jolla, CA 92093, USA; 2 VA San Diego Healthcare System, 3350 La Jolla Village Drive, La Jolla, CA 92151, USA; 3 Institute for Genomic Medicine, University of California San Diego, La Jolla, CA 92093, USA

**Keywords:** Bipolar Disorder, Pharmacogenomics, Lithium, Antidepressants, Antipsychotics

## Abstract

**Background:**

Bipolar disorder (BD) is a psychiatric illness defined by pathological alterations between the mood states of mania and depression, causing disability, imposing healthcare costs and elevating the risk of suicide. Although effective treatments for BD exist, variability in outcomes leads to a large number of treatment failures, typically followed by a trial and error process of medication switches that can take years. Pharmacogenetic testing (PGT), by tailoring drug choice to an individual, may personalize and expedite treatment so as to identify more rapidly medications well suited to individual BD patients.

**Discussion:**

A number of associations have been made in BD between medication response phenotypes and specific genetic markers. However, to date clinical adoption of PGT has been limited, often citing questions that must be answered before it can be widely utilized. These include: What are the requirements of supporting evidence? How large is a clinically relevant effect? What degree of specificity and sensitivity are required? Does a given marker influence decision making and have clinical utility? In many cases, the answers to these questions remain unknown, and ultimately, the question of whether PGT is valid and useful must be determined empirically. Towards this aim, we have reviewed the literature and selected drug-genotype associations with the strongest evidence for utility in BD.

**Summary:**

Based upon these findings, we propose a preliminary panel for use in PGT, and a method by which the results of a PGT panel can be integrated for clinical interpretation. Finally, we argue that based on the sufficiency of accumulated evidence, PGT implementation studies are now warranted. We propose and discuss the design for a randomized clinical trial to test the use of PGT in the treatment of BD.

## Background

Bipolar disorder (BD) is a psychiatric illness that affects 3% to 5% of the population [1], and is characterized by recurring and severe changes in mood, leading to significant impairment, and increased risk for suicide. The estimated direct and indirect costs of BD in the US are estimated at $15B [2]. Mood stabilizers are considered the treatment of choice for managing BD. However, evidenced-based treatment guidelines include a wide range of medications, such as antipsychotics, antidepressants, anxiolytics and multi-drug combinations [3,4]. Despite the help of guidelines, psychiatrists face tremendous challenges in treating BD. First, the presentation of BD is heterogeneous, and the diagnosis of BD is often difficult. Psychiatric co-morbidity is common, and the typical patient with BD is misdiagnosed an average of four times [5]. After the diagnosis is established, treatment response is often inadequate, and the rate of remission is poor, particularly among those in the depressive phase [6]. Finally, many drugs are poorly tolerated, and unwanted medication side effects are common. Therefore, while there are broadly accepted best practices for treating BD, their application yields variable, and often unacceptable, outcomes, leaving many patients to suffer prolonged and unsuccessful drug trials before responding to treatment.

A challenge in treating BD is heterogeneity among patient responses. The same drug may have different outcomes in two otherwise similar BD patients, successfully treating one, but necessitating a search for an alternative treatment in the other. For this reason, a typical course of treatment is extensive, consisting of serial medication trials until an effective drug or drug combination is identified. Variability in treatment responses may be related to differences in illness pathophysiology and drug metabolism, both factors thought to be influenced by an individual’s genetic background. Therefore, understanding the relationship between genetic factors and treatment response may allow for the development of objective pharmacogenetic tests (PGT) to guide the physician rationally and rapidly towards effective drug treatments for BD.

Recently, many genetic variants have been associated with response-related psychiatric phenotypes, including many in BD. While the strength of this evidence varies, some are now considered reliable. Despite this progress, implementation of this knowledge in the clinic has been limited. It remains unclear if association to response will necessarily translate into improved outcomes, and there has been a reluctance to apply these findings clinically. Other medical disciplines have increasingly demonstrated the utility of personalized medicine [7] with PGT showing advantages in warfarin dosing [8], thiopurine myelosuppression in leukemia [9,10] and abacavir hypersensitivity in HIV [11,12]. However, these fields are experiencing similar inertia, even in cases where the supporting evidence is strong [13].

Questions have been raised in the psychiatric literature regarding the appropriateness of implementing PGT in the clinic, most of which remain unanswered [14]. However, unlike genetic association studies which cannot address clinical utility, implementation studies may provide empirical answers to some of these pertinent issues [Box 1]. Hence, they are urgently needed. In this opinion, we conclude that a few PGT markers, while perhaps not perfect are sufficiently well-established to advocate for research into their implementation to determine if they have clinical value. A preliminary path forward and discussion of the anticipated benefits and obstacles is presented.

## Discussion

### Pharmacogenetic association studies

A large number of genetic markers have been associated with psychotropic response in psychiatry, including many relevant to BD. A number of reviews have been published in recent years [14,15], focused primarily on lithium. However, many drugs used to treat major depression (MDD) or schizophrenia (SCZ) are also effective in BD. Therefore, in organizing this opinion around implementation, we have broadened the scope of PGT in BD to consider these options in addition to lithium. PubMed and the PharmGKB [16] database were searched for pharmacogenetic reports for lithium, anti-seizure drugs, antipsychotics and antidepressants. We then devised criteria to rank these results based on strength of evidence and clinical utility. In doing so, we focused on variants that have been independently replicated in large cohorts. In some cases, the studies were performed in MDD or SCZ. We developed standardized criteria by which genetic markers were assessed. Those categorized as evidence Level 1 were replicated at least once with a *P*-value of <0.05 after correction for multiple comparisons, in a total population of ≥500 cases of similar ethnicity. Level 2 evidence required replication with *P*-values <0.05 after correction and at least one population of 100 to 499. Studies with *P*-values <0.05, but without replication, or in samples <100 were categorized as Level 3. Our search yielded a set of 16 genetic markers that were classified as Level 1 (Table 1). We also included a small number of variants that were compelling candidates based upon convergent animal or pre-clinical studies that met the threshold for Level 2 evidence. Each genotype-drug association is discussed below.

**Table 1:** Genetic variants with evidence supporting clinical utility

**Drug**	**Gene**	**Marker**
Lithium	BDNF	rs6265
	NTRK2	rs1387923
	CREB1	rs6740584
	GRIA2	rs9784453
	GSK3B	rs1954787
Carbamazepine	HLA	rs2844682; rs3909184
Antipsychotics	DRD2	rs1799732
	HTR2A	rs6311
	ANNK1	rs1800497
	HTR2C	rs3813929
SSRI’s	GRIK4	rs1954787
	HTR2A	rs7997012
	SLC6A4	5-HTTLPR
		rs25531
Drug Metabolism	CYP2D6	Multiple markers [124-126]
	CYP2C19	Multiple markers [115,151-153]

SSRI’s, selective serotonin receptor inhibitors.

### Pharmacodynamics

#### ***Lithium response***

Lithium is the most widely used mood stabilizer, greatly decreasing suicide risk and BD symptoms during acute mania, depression and maintenance [17,18]. Clinical features have been associated with lithium response [19,20], leading some to argue that lithium responsive BD is a distinct illness subtype that can be defined in part by its sensitivity to the drug [21]. However, in a long term prospective study [22], while lithium response was identified as an enduring trait, the classical clinical profile failed to discriminate lithium responders from non-responders. For this reason, efforts have been directed towards pharmacogenetic approaches predicting lithium response. Table 2 summarizes some of the genetic markers reported to be associated to mood stabilizer response.

**Table 2:** Genetic markers associated with mood stabilizer outcomes

**Drug/Phenotype**	**Gene**	**Marker**	**Subjects**	**Studies**	**Diagnosis**	**Phenotype association**	**Level of evidence**
Lithium	BDNF	rs6265	538	3	BD I/II	A(Met)-allele associated with better response	1
	NTRK2	rs1387923	284	1	BDI	T-allele associated with better response	2
	CREB1	rs6740584	258	1	BD I/II	GA genotype associated with better response	2
	GRIA2	rs9784453	817	2	BD I/II	A-allele associated with worse response	1
	ODZ4	rs11237637	817	2	BD I/II	T-allele associated with worse response	1
	GSK3B	rs1954787	307	3	BD I	C-allele associated with better response	2
Carbamazepine	HLA	HLA-B*1502	380	2	Unspecified	HLA-B*1502 associated with SJS/TEN	2

BD, bipolar disorder; SJS, Stevens-Johnson syndrome; TEN, toxic epidermal necrolysis.

#### ***BDNF and NTRK2***

Brain-derived neurotrophic factor (BDNF), plays a crucial role in neuronal survival, differentiation and plasticity in the brain [23-25]. BDNF binds to the TrkB receptor, the protein product of *NTRK2*. The BDNF/TrkB signaling pathway is involved in a wide range of intracellular signaling cascades [26] and is thought to play an important role in mediating the therapeutic effects of lithium [27,28]. Past evidence has implicated BDNF in the pathogenesis of BD [29-31], and the *BDNF*^Val66Met^ polymorphism (rs6265) has been associated with lithium response, whereby carriers of the Met allele had better treatment outcomes [32,33]. The lithium response association with BDNF was recently replicated in a sample of Chinese patients with BD [34], but others have failed to replicate the result [35]. *NTRK2* polymorphisms have been implicated as genetic factors underlying BD in a genomewide association study (GWAS) [36] and associated with lithium response in two independent studies. In the first, two SNPs in *NTRK2* were associated with response specifically in patients with euphoric mania [37]. This association has been subsequently replicated in an independent prospective trial [38]. More recently, another independent study again implicated *NTRK2,* using a different variant [39]. However, not all studies have replicated the *NTRK2* association with lithium response [32]. Different variants in NTRK2 have been associated with risk for suicide attempts, which is particularly interesting in the light of lithium’s known anti-suicide properties [40].

#### ***CREB***

The cAMP response element-binding protein (CREB1) is a transcription factor that plays important roles in neuroplasticity, cell survival and neuronal modulation by lithium and antidepressants [41-43]. An association between variants in *CREB1* and lithium response was reported in a prospective association study conducted on a sample of 258 subjects followed over three years, in which the BD subjects were treated with lithium monotherapy [44]. Of interest, independent *CREB1* haplotypes have been associated with selective serotonin receptor inhibitor (SSRI) remission [45], emergent suicidal ideation during SSRI treatment [46] and treatment resistant depression [47], features previously linked to latent bipolarity among depressed subjects.

#### ***GRIA2, ODZ4/TENM4***

In a GWAS of lithium response conducted using subjects from the Systematic Treatment Enhancement Program for Bipolar Disorder (STEP-BD), five SNPs showed association with *P* values of <5 × 10ˉ^4^ and were independently replicated in a cohort of 359 BD patients. Among these was a variant in *GRIA2,* a glutamate receptor subunit gene [48]. Glutamate has been strongly implicated in the pathogenesis of BD, and *GRIA2* was previously shown to be regulated by lithium in hippocampal neurons [49]. *ODZ4* (also named *TENM4*), has been associated with BD susceptibility in the largest published GWAS to date and was also found to be associated with lithium response [50,51].

#### ***GSK3B***

Glycogen synthase kinase 3β (GSK3B), is an enzyme involved in neuronal development and survival, and is inhibited by lithium [52,53]. A pharmacogenetic study of 88 BD patients identified a functional SNP associated with lithium response in the *GSK3B* promoter [54]. Similar results were obtained in a cohort of 138 Taiwanese BD subjects [55] and a mixed cohort of BD/MDD receiving augmentation of antidepressants with lithium [56]. However, other studies have failed to detect a similar association [57,58].

#### ***Carbamazepine response***

The anticonvulsant carbamazepine (CBZ) is a mainstay of treatment in BD patients [59,60]; however, no pharmacogenetic studies to date have evaluated response to carbamazepine. Clinical features associated with CBZ response include those with BPII disorder, dysphoric mania, comorbid substance abuse, mood incongruent delusions, negative family history of BD in first-degree relatives, and in those not tolerant to other treatments due to side effects (for example, weight gain, tremor, diabetes insipidus, or polycystic ovarian syndrome) [61].

There has been a strong association reported between the HLA-B*1502 haplotype and the severe life-threatening cutaneous drug reactions, Stevens-Johnson syndrome (SJS) and toxic epidermal necrolysis (TEN) [62]. Two studies conducted on Han Chinese patients reported that HLA-B*1502 was present in 100% (44/44) and 98.3% (59/60) of CBZ-induced SJS patients in contrast to 3% (3/101) and 4.2% (6/144) of CBZ-tolerant patients [63,64]. While the risk-associated haplotype is found in 5% to 15% of Asians, clinical guidelines in some healthcare institutions suggest avoiding carbamazepine in all patients of Asian background, thereby potentially depriving the majority of patients in this racial group a proven treatment for BD. Hence, the adaptation of PGT for *HLA-B* has a huge potential for impact in this group and *HLA-B* PGT for carbamazepine treatment is now recommended by the Clinical Pharmacogenetics Implementation Consortium (CPIC) [65].

#### ***Valproic acid response***

Relatively little work has been done on the pharmacogenetic response to the anticonvulsant mood stabilizer, valproic acid, and no valproic acid study meets the requirements for inclusion in our implementation list. However, valproic acid is a first line treatment for BD, and its omission constitutes an important gap. Valproic acid pharmacogenetics is likely to be a priority for future research.

#### ***Antipsychotic response***

Antipsychotics are commonly used in BD, both as a first-line treatment in acute mania [66] and depression [67]. However, psychiatrists are increasingly reluctant to use them due to their unfavorable long-term side effect profile, especially weight gain [68]. In addition to metabolic problems, the risk of extra-pyramidal side effects (EPS) associated with second generation antipsychotics is estimated to be 13% to 17% and remains a concern.

Because the majority of the pharmacogenetic studies of antipsychotics have been done with SCZ patients, it requires extrapolation to BD. For this reason, caution is required in interpreting these results. However, many of these data likely apply to BD. First, there is substantial symptom overlap and shared genetic risk between SCZ and BD [50,69], suggesting that underlying illness mechanisms are shared. Moreover, because side effects such as weight gain are assumed to be due to ‘off target’ effects, side effect mechanisms are likely similar across disorders. To be sure, research extending PGT of antipsychotics to BD is required, not only with regard to psychotic symptoms, but also with respect to mood. Genetic variants reported to be associated with antipsychotic response are listed in Table 3.

**Table 3:** Genetic markers associated with antipsychotics outcomes

**Drug/Phenotype**	**Gene**	**Marker**	**Subjects**	**Studies**	**Diagnosis**	**Phenotypic association**	**Level of evidence**
Antipsychotics	DRD2	rs1799732	687	6^a^	Schizophrenia	Del-allele associated with poor response	1
	HTR2A	rs6311	315	2	Schizophrenia	A-allele associated with good response	2
	ANNK1	rs1800497	1,256	6^a^	Schizophrenia	A2-allele associated with TD risk	1
	HTR2C	rs3813929	1,108	14^a^	Schizophrenia	C-allele associated with weight gain	1
	MC4R	rs489693	344	4	Unspecified	AA genotype associated with weight gain	2
		rs17782313	345	1	Multiple	C-allele associated with weight gain	2

^a^Includes meta-analyses. TD, tardive dyskinesia.

#### ***DRD2/ANKK1***

The D2 dopamine receptor encoded from *DRD2* is the primary target of most antipsychotic medications [70,71]. The -141C insertion/deletion polymorphism located in the *DRD2* promoter, has been shown to have a functional effect on expression and has been studied on several occasions with respect to antipsychotic outcomes. In a meta-analysis of 687 SCZ patients from six studies, the -141C variant demonstrated an association with favorable antipsychotic response (>50% reduction in symptoms) at eight weeks [72]. The ankyrin repeat and kinase domain containing 1 gene (*ANKK1*) is located 10 kb from *DRD2 *[73], and has been associated with tardive dyskinesia (TD). In a meta-analysis of 1,256 SCZ subjects from six studies, *ANKK1* genotype was associated with TD, with odds ratios of 1.30 to 1.50 [74]. A decrease in *DRD2* expression was also associated with a risk conferring allele [75,76], perhaps explaining the effect of *ANKK1* genotype on TD risk [77].

#### ***HTR2A***

Many antipsychotic drugs alter serotonin signaling by blocking 5HT2A receptors, encoded by *HTR2A *[78]. Several studies demonstrated a less favorable response to olanzapine and clozapine among carriers of the rare functional promoter variant, A-1438G in *HTR2A *[79-81].

#### ***HTR2C***

In attempts to identify genetic vulnerability to antipsychotic induced weight gain and metabolic syndrome, *HTR2C*, encoding the 5-HT2C receptor, has yielded the most reliable associations. A meta-analysis of eight studies showed a significant association of C759T *HTR2C* SNP, with a lower risk of weight gain with antipsychotics [82]. Similarly, ten independent studies on schizophrenic patients have shown a significant association between the C-allele of the same SNP and higher risk of antipsychotic-induced weight gain [77].

#### ***MC4R***

*MC4R*, one of the most important genes associated with weight gain and increased risk for obesity [83,84], has been shown to play a role in the modulation of food intake and energy homeostasis [85,86]. It has also been associated with atypical antipsychotics-induced weight gain in a GWAS conducted on 139 pediatric subjects, with replication in three additional cohorts [87]. Another SNP, previously associated with weight gain [83] yielded similar results [88].

#### ***Antidepressant response***

Although controversial [89], SSRIs are widely used in conjunction with mood stabilizers or antipsychotics for depression in BD, appearing in evidence-based psychopharmacology guidelines [90,91]. However, because of their questionable efficacy and potential for inducing mania, there is a need to identify genetic variants associated with response and adverse events, such as manic switch and rapid cycling. SSRI response has been primarily studied in MDD or mixed BD/MDD samples. Therefore, our extrapolation from MDD to BD may have important caveats in the use of these markers. Table 4 summarizes genes associated with antidepressant response.

**Table 4:** Genetic markers associated with antidepressant response

**Drug/Phenotype**	**Gene**	**Marker**	**Subjects**	**Studies**	**Diagnosis**	**Phenotype association**	**Level of evidence**
Antidepressant	GRIK4	rs1954787	1,816	2	MDD	CC genotype associated with better citalopram response	1
	HTR2A	rs7997012	1,329	1	MDD	A-allele associated with better citalopram response	1
	SLC6A4	5-HTTLPR	1,435	15	MDD, BD	long allele associated with better SSRI response, short allele increases risk of AIM	1
	FKBP5	rs4713916	1,426	4	MDD, BD, dysthymia	A-allele associated with better antidepressant response	1
	ABCB1	rs2032583, rs2235040	689	2	MDD	C and A alleles respectively associated with better response	1
			424	1	MDD	C and A alleles respectively associated with adverse effects	2

AIM, antidepressant induced mania; BD, bipolar disorder; MDD, major depression; SSRI, selective serotonin receptor inhibitor.

#### ***SLC6A4 (5-HTTLPR)***

The *5-HTTLPR* is a 44 bp insertion/deletion within the promoter of *SLC6A4*, the gene encoding the serotonin transporter [92]. Transcriptional activity among carriers of the short allele (S) has been shown to be lower compared to carriers of the long allele (L) [92,93]. Fifteen studies encompassing 1,435 MDD and BD patients were analyzed for association with SSRI response [94]. The L variant was associated with a better response, while SS genotype was linked to lower rates of remission and longer response times. BD patients carrying the S allele were also more susceptible to developing anti-depressant-induced mania (AIM) with a 35% increase in risk [95].

#### ***GRIK4***

The Sequenced Treatment Alternatives to Relieve Depression (STAR*D) study included an initial period of citalopram monotherapy. Of these SSRI-treated subjects, 1,816 were genotyped in a candidate gene study of 768 SNPs, identifying an association between the *GRIK4* SNP rs1954787 and therapeutic response [96]. *GRIK4* is a component of the ionotropic kainate/glutamate receptor. An attempt to replicate this finding in 387 BD/MDD subjects found supportive evidence for a *GRIK4* association, but not at the same SNP, and required a gene x gene interaction [97].

#### ***HTR2A***

Since the serotonin transporter is a direct target of SSRIs, modulation of serotonin transmission has long been thought a key mechanism of action. A number of serotonin receptors have been studied and, in the STAR*D sample, an analysis of 1,953 patients revealed a strong association between the intronic *HTR2A* SNP rs7997012 and treatment response [98]. In a synergistic manner, homozygous carriers of the protective alleles of both *GRIK4* and *HTR2A* were 23% more likely to respond to citalopram than participants carrying neither of these alleles [96].

#### ***FKBP5***

*FKBP5* has been implicated in psychiatric disorders because of its role encoding a co-chaperone protein for the glucocorticoid receptor. It has been shown to affect the hypothalamic–pituitary–adrenal (HPA) [99,100] and modulate Akt activity [101], thereby altering numerous neuronal functions [102,103]. Variation in *FKBP5* was first associated with antidepressant response in two independent samples [104]. In a subsequent meta-analysis of eight studies including more than 2,199 patients, this association was replicated [105].

### Genome-wide association studies

Because the results have been better replicated, most of the markers discussed above come from candidate gene studies. However, by surveying the entire genome, GWAS have the ability to identify previously unrecognized pharmacogenetic markers that could not be predicted based on candidate hypotheses. While some of these studies require replication or have failed to achieve genome-wide significance, several large GWAS of drug response have been conducted and are worthy of mention.

A GWAS of lithium response recently reported very strong evidence of association (*P* = 10^-37^) for a variant in the gene glutamic acid decarboxylase like 1 (*GADL1*) in a sample of 294 Taiwanese subjects [106]. This result was then replicated in smaller samples of 100 and 24 subjects by the same investigators. This is an exciting result awaiting independent replication. A GWAS analysis of citalopram response in the STAR*D cohort yielded suggestive associations for markers in *RORA*, *UBE3C* and *BMP7*, but none that met genome wide thresholds for significance [107]. A similar analysis revealed a number of suggestive associations of citalopram-induced side effects, most prominently a marker in *EMID2*, associated with altered vision and hearing [108]. More recently, data emerged from a meta-analysis of >2,200 MDD patients from the STAR*D, Genome-Based Therapeutic Drugs for Depression (GENDP) and Munich Antidepressant Response Signature (MARS) trials [109]. In this study, a number of suggestive markers were associated with various response indicators, but none met genome-wide thresholds for significance*.* Taking a different approach, GWAS analysis for copy number variation using the STAR*D sample and a health system repository revealed a modest enrichment of chromosomal duplications among treatment refractory depression cases, and a deletion in *PABPC4L* uniquely affecting treatment resistant MDD patients, but was restricted to only a small number of subjects [110].

As with case–control studies of BD, MDD and SCZ, GWAS approaches have generally not supported previously reported candidate gene associations. While the reasons for this discrepancy are unclear, it does underscore the relative strengths and weaknesses of candidate gene studies and GWAS. Candidate gene studies are limited in scope, focusing on individual genes, while GWAS surveys the entire genome. Yet because of this focus, candidate gene studies often have greater statistical power with smaller sample sizes for any individual gene. Practically, however, these studies do not generally correct for the total number of SNPs examined in the same sample. The price of the wide scope of GWAS is the large number of statistical tests and the very large sample sizes required to achieve statistical significance. Such large sample sizes are particularly problematic for pharmacogenetics where the cost of assessing the phenotype, via a prospective clinical trial, is very high, and uniformity of treatment among subjects is rare. Future GWAS with larger samples will likely identify many new drug response loci. Until then, we argue that the strongest candidate gene markers likely provide valid, but incomplete, answers that warrant testing for utility.

### Pharmacokinetics

#### ***CYP450 enzymes***

Cytochrome P450 enzymes constitute a major component of the human drug metabolism system, activating/deactivating and facilitating the elimination of compounds [111,112]. Many drugs used to treat BD are eliminated by CYP450 enzymes, and knowledge of a patient’s CYP450 genotype can be invaluable in selecting medications or doses. While pharmacokinetics have been largely neglected in previous reviews of the pharmacogenetics of BD, CYP450 genotyping is now approaching mainstream use, with FDA approval of CYP450 testing for 27 alleles in *CYP2D6* and three alleles in *CYP2C19 *[113,114]. CYP450 genotypes are highly variable, with numerous functionally distinct haplotypes, but are frequently organized by metabolic activity into different phenotypic categories. For example, 2D6 phenotypes are commonly classified as: poor metabolizers (PM) with completely absent enzymatic activity; intermediate metabolizers (IM) with decreased enzymatic activity; extensive metabolizers (EM) with normal enzymatic activity; and ultra-rapid metabolizers (UM) with increased enzymatic activity [115]. The relative frequency of minor allelic variants differs greatly by ethnicity [116-118]. For example, among East Asians approximately 30% carry the null *CYP2C19* haplotypes *2 and *3, that lead to a total absence of CYP2C19 activity. In contrast, the frequency in Caucasians ranges from 4% to 13% [119]. On the other hand, <5% of East Asians possesses the *CYP2C19* *17 allele that increases activity, whereas the rate in Northern European Caucasians is 18% [120,121].

#### ***CYP2D6***

CYP2D6 is involved in the metabolism of more than 70 drugs, including many anti-depressants and anti-psychotics. The *CYP2D6* gene is located at 22q13.1, and encodes the CYP2D6 enzyme [122,123].

#### ***CYP2D6 and antidepressants***

*CYP2D6* genotype has been shown to be associated with antidepressant outcomes across several dimensions [124-126]. Response to venlafaxine was significantly greater in *CYP2D6* EM subjects who metabolize the drug normally compared IM subjects who require lower doses [127]. MDD patients showed higher rates of remission among *CYP2D6* IMs compared to PMs, after eight weeks of escitalopram treatment [128]. *CYP2D6* UM status contributed to non-response by increasing early dropout rates [129,130], and *CYP2D6* UM status was associated with a higher risk of suicide [131-133]. Finally, *CYP2D6* genotype predicts the risk of adverse effects as shown among PMs treated with venlafaxine and tricyclic antidepressants (TCAs) [134,135]. Most dramatically, cases have been reported of PMs being linked to fatal responses to anti-depressants [136,137]. While compelling evidence links functional *CYP2D6* haplotypes to drug levels [138], other studies failed to determine an association between *CYP2D6* polymorphisms and treatment response [139-141], leading the Evaluation of Genomic Applications in Practice and Prevention (EGAPP) group to conclude that the evidence in support of *CYP2D6* genotyping for guiding antidepressant treatment is inconclusive, and recommending implementation clinical trials in order to show the benefits from CYP450 genotyping [142].

#### ***CYP2D6 and antipsychotics***

CYP2D6 mediates the metabolism of many antipsychotics, making the use of genetic information about this locus a rational strategy for personalized medicine [143-146]. However the utility of *CYP2D6* genotyping in predicting clinical response to antipsychotics is relatively unexplored. *CYP2D6* genotype did predict tolerability to risperidone with a higher risk of adverse effects among PMs [147,148].

#### ***CYP2C19***

CYP2C19 is involved in the metabolism of a wide range of anti-depressants and benzodiazepines [149,150]. The *CYP2C19* gene is located at 10q24.1-q24.3.

#### ***CYP2C19 and antidepressants***

CYP2C19 has been shown to be the primary metabolic enzyme for citalopram, escitalopram, amitriptyline and clomipramine [115,151-153]. Functional *CYP2C19* haplotypes have been associated with serum concentrations of citalopram [151,154] and escitalopram [138,155]. Analysis of the Caucasian subset of the STAR*D sample revealed an association between the inactive *2 allele and tolerance to citalopram [156].

### Other pharmacokinetic targets

#### ***ABCB1***

P-glycoprotein (P-gp), one of the ATP-binding cassette (ABC) transporter proteins, actively transports drugs, including antidepressants, across the blood–brain barrier [157,158]. Multiple SNPs in *ABCB1,* coding for P-gp, were associated with response to citalopram, paroxetine, amitriptyline and venlafaxine*,* substrates of P-gp [159]. Two of the latter were replicated in a geriatric sample receiving paroxetine [160] and associated with SSRI-related adverse effects in a separate study [161].

### Pharmacogenetics and clinical practice: are we there yet?

Psychiatry lacks objective biological disease markers to guide diagnosis and treatment, creating a situation analogous to cardiologists relying on subjective descriptions of chest pain, without the aid of electrocardiograms, biochemical markers, or blood pressure cuffs to differentiate among syndromes or track recovery. While the discovery of predictive genetic markers is just beginning, evidence already exists for a number of well replicated PGT markers. This has raised a number of questions in the psychiatric literature [162], including: How strong must the supporting evidence be? How large is a clinically relevant effect? What degree of specificity and sensitivity are required? Does a given marker influence decision making and have clinical utility? In short, can the existing genetic data be translated from statistical associations to clinically informative tools? The answer remains unclear. A genetic marker can correlate with an outcome, but be deemed clinically useless because of low predictive value, poor specificity or limited external validity [163,164]. Some have argued against implementation [162-165], concluding that PGT markers are not yet sufficiently sensitive or specific [165]. However, the utility or lack of utility of PGT is an empirical question that has remained largely untested.

### Pharmacogenetic implementation studies: design and execution

**Figure 1:**
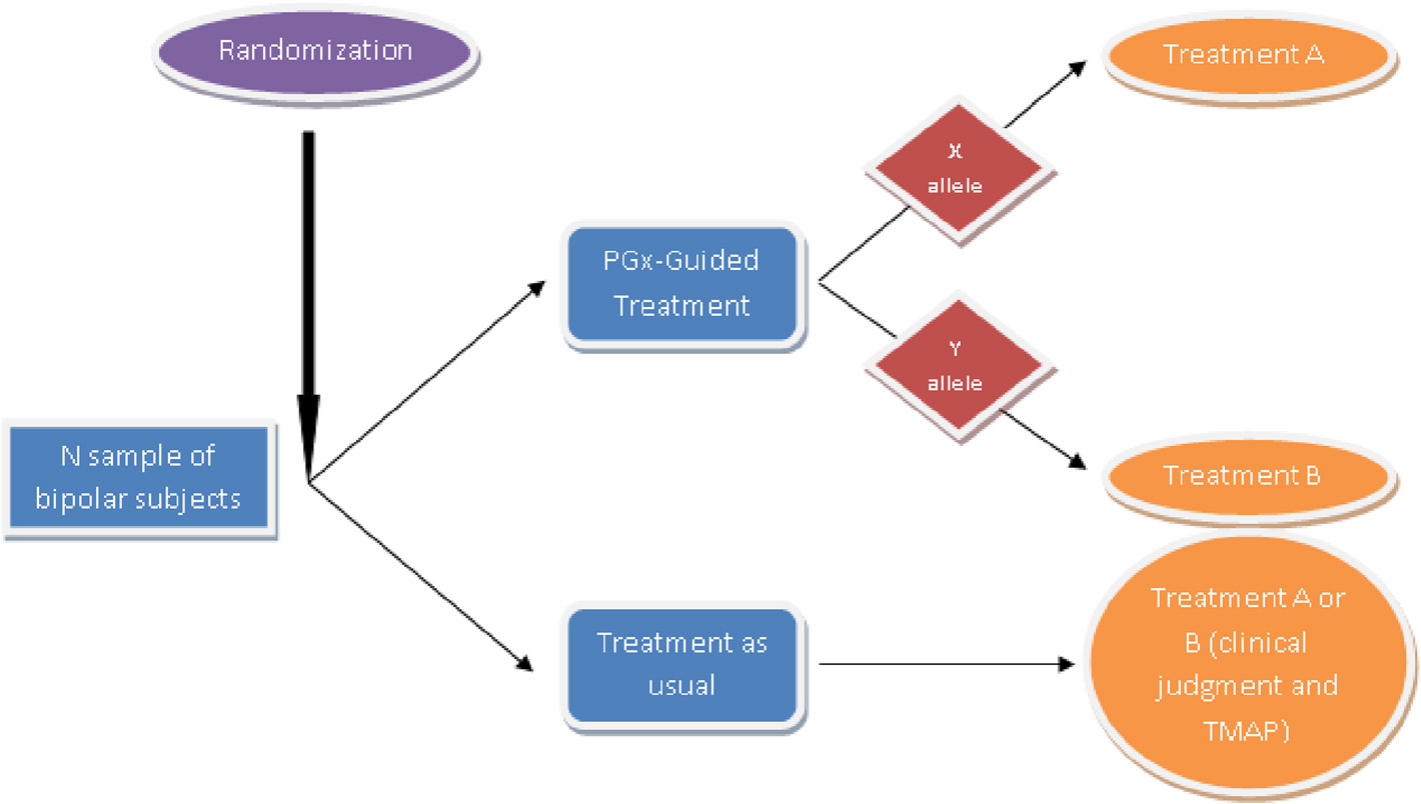
**A pharmacogenetics implementation design.** Patients are randomized to pharmacogenetic test (PGT) guided treatment or treatment as usual (TAU). For the PGT group, the physician incorporates the results of the test to make treatment decisions; in the TAU group, the physician treats according to usual practice based on evidence-based treatment guidelines. Subjects are assessed longitudinally and outcome compared after the specified treatment interval.

The gold standard for determining the utility of a therapeutic intervention is the randomized clinical trial. This method is readily applied to PGT by comparing the outcomes of PGT guided treatment against treatment as usual (TAU). Figure 1 illustrates a simple, two arm design that could test the clinical utility of PGT. Outcomes could be measured across a number of dimensions including response magnitude, time to response, symptom burden, side effect burden, disability and cost. In principle, improvement on even a single dimension could constitute a successful trial.

### Designing the test panel

The design of PGT panels is likely to evolve rapidly. Costs for whole genome sequencing are falling rapidly, and it is expected that this technology will be widely available once the cost drops below $1,000 [166]. Therefore, in the near future, costs of whole genome sequencing will be comparable to other common medical tests, allowing for the patient’s entire genetic sequence and interpretation to be part of the electronic medical record. In the interim, candidate gene panels composed of a limited set of markers, targeting key biological functions offer a cost effective and technically plausible format for focused genotyping. For illustrative purposes, we have selected the well replicated markers described above to show how a candidate gene variant panel might be selected, configured and implemented.

### Reporting and recording genetic results

Most psychiatrists lack training in advanced genetics and may have difficulty understanding PGT results. Therefore, in the design of a PGT, it is important to convey results in a simple manner that can be understood and quickly incorporated into clinical decision making without losing pertinent detail. To address the dilemma presented by these competing interests, a PGT report may require multiple layers, each coherent and internally consistent, that presents information with increasing levels of detail, starting with general drug recommendations and ending with the raw genotype results and technical details of the assay.

### An algorithm for integrating genetic results

**Figure 2:**
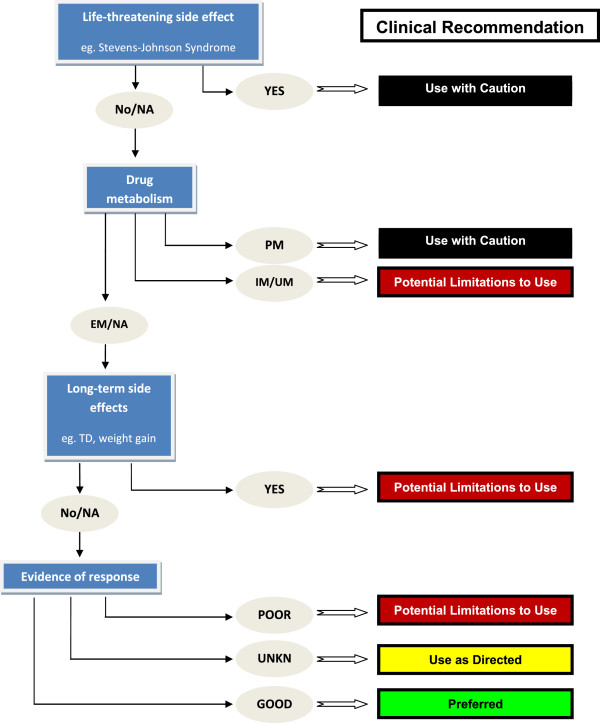
**Integrating pharmacogenetic test results.** An algorithm for translating genotypes into specific recommendations for drugs commonly used in BD is illustrated. In making an overall treatment recommendation, all possible drug-genotype combinations are classified into four outcome categories. The overall recommendation is optimized to avoid the worst outcome predicted by PGT. Drug-genotype combinations associated with serious and/or potentially life threatening outcomes are given lowest priority (Use with caution). Drug-genotype combinations with an elevated risk of long term side effects or that are predicted to require higher dosing requirements are given the next lowest priority (Potential limitations to use). Drug-genotype combinations that are not associated with an increase in adverse events are recommended for use in accordance with standard practices (Use as directed), and those without an elevated risk for adverse events, and an association with good psychiatric outcomes are given highest priority (Preferential use). EM, extensive metabolizer; IM, intermediate metabolizer; NA, not available; PM, poor metabolizer; TD, tardive dyskinesia; UM, ultra-rapid metabolizer; UNKN: unknown.

Frequently, a medication may have multiple, relevant genetic marker associations (for example, one for response, others for side effects), a genetic marker may be relevant to multiple medications, and different genetic markers may suggest opposite actions. Therefore, the PGT results for each variant need to be reconciled and consolidated into a single, overall recommendation. The process of synthesizing PGT results from multiple markers into coherent treatment recommendations for the physician may prove challenging. One solution to this challenge is to develop algorithms that prioritize and apply differential weight to potential clinical outcomes (Figure 2). We approached this by first classifying all relevant drug-marker combinations into four clinical recommendations: 1) use with caution; 2) potential limitations to use; 3) use as directed; and 4) preferred use. We then developed an algorithm (Figure 2), that generates an overall recommendation for each drug based on genotype. In this algorithm, drugs are serially considered in descending order of expected adverse events, starting with the most severe. For example, genotypes that predict high risk for life-threatening/serious adverse effects are considered first, thereby removing drugs that could present imminent danger to the subject (for example, HLA-B1502 and SJS). Medications are then considered with respect to genetic risk of long-term side effects and/or higher dosing requirements (for example, CYP450 UMs). Finally, drugs are assessed in terms of genetic associations with symptom response. The set of drugs with minimal side effect associations, but no information on response will be classified for ‘Use as directed’. Those with minimal/no side effect associations predicted to show a good response are classified for ‘Preferred use’. Given the common nature of many of the risk-associated markers that preclude ‘preferred’ status, this latter set of drugs will typically be small, (perhaps even nonexistent for patients with some genotypes), but optimally matched to an individual, narrowing the available choices for making ‘the best decision’, and eliminating choices that are less likely to work or to be poorly tolerated. Importantly, the algorithm does not replace clinical judgment, and the physician is free to incorporate relevant information from other sources (for example, clinical features, drug-drug interactions) to weigh options alternatively, or, when indicated, to override the algorithm.

### Special considerations in clinical trial design

There are several factors that differ between a PGT implementation study and a randomized clinical trial (for example, for a medication) or genetic association study that warrant special consideration. First, in designing an implementation study, a panel of genetic markers is evaluated, not a single genetic marker, and the efficacy of the panel as a whole is tested. Not all markers will be relevant to a given patient or decision, but the breadth of multi-marker panels affords the clinician an opportunity to survey several low frequency alleles with good efficiency, offering broad utility in a naturalistic environment. This approach does have limited ability to test the benefit of any single genetic association included in the panel. It is assumed that not every result will support a previously established genetic association, but that in aggregate, treatment outcomes will be improved based on the PGT results. Over time, data can be collected regarding clinician choices and specific markers. In this manner, insight into which components of the test are most useful can be developed.

In an implementation study, the effectiveness of treatment, regardless of the specific drug(s) used is the most important variable. The hypothesis is that outcomes in PGT are superior to TAU as a result of genetic information being conveyed to the physician, not that a specific drug is better. Appropriate clinical use of PGT data will differ among clinicians, and this variability must be controlled. The use of evidence-supported clinical treatment guidelines can standardize some practices. However, ‘real world patients’, particularly those with treatment refractory symptoms, often have extensive treatment histories that can make it difficult to fit them into standardized algorithms. To mitigate this difficulty, experimental blinding is essential. Since the physician is required to interpret genetic data and make decisions based upon the results, the physician cannot be blinded. However, by obtaining DNA on all subjects, patients can be blinded if their data are withheld. Independent raters can also be blinded to assess outcomes. Many other clinical trial designs are possible, and their respective strengths and weaknesses have been recently reviewed [162].

### Early implementation studies

Using a panel of five genetic markers and a design similar to the one outlined above, a PGT implementation study of 44 MDD patients was completed using a commercially available test, yielding promising results [167]. Treatment refractory patients with MDD were divided into PGT and TAU groups. Depressive symptoms were significantly decreased in the PGT group compared to TAU, as shown by greater reductions in QIDS-C16 and HAM-D17 scores at the eighth week. More recently, similar differences were shown by the same group using a larger replication sample of 227 MDD patients [168]. The same test was retrospectively applied to 97 subjects for whom health insurance records were available. In this way, genetic testing predicted healthcare utilization and costs. When patients were prescribed medications later found to be poorly matched to genotype, they required more frequent visits, took more medication and required greater expenditures [169]. In all three of these reports, differences in outcome were driven primarily by the genotypes expected to have the most severe outcomes, primarily those expected to metabolize medications poorly, resulting in more adverse effects. Strikingly, these most severe mismatches were commonly encountered, affecting approximately 25% of the subjects in one study [168]. Several limitations in these studies warrant mention. First, both efficacy trials were open label and not randomized, meaning that patients were aware of their group assignment. Second, the samples were small, ethnically homogenous and collected from single sites, potentially limiting the external validity of the results. Nonetheless, the improvement over TAU is particularly striking given the nature of the subjects, many of whom suffered from chronic refractory depression. To date, no trials in BD have been conducted.

## Summary

### Statistically significant, but clinically significant?

The findings summarized indicate that a number of PGT markers are reliably associated with clinically salient treatment outcomes. Although this is a very early stage in the discovery of PGT markers in BD, this set of genes is expected to be refined and enlarged over time, and we argue that there are already enough potentially informative results to warrant implementation studies to determine their clinical utility.

Whether or not the pharmacogenetic panel presented here can be used to improve outcomes in clinical practice is an untested question. In clinical trials, the concepts of effect size, sensitivity (labeling a true positive as positive) and specificity (labeling a true negative as negative) are used to measure the value of a biological test. Psychiatric PGT has been criticized for individual allelic associations falling short on these measures. However, three counter-points can be offered. First, PGT implementation takes advantage of multiple genetic markers, and while individual markers may be non-specific, limited in applicability, or yield small effects, a more comprehensive panel, utilizing multiple markers may prove to be more robust. Second, in the absence of objective markers to guide decisions, medication choice in BD is presently made in a largely trial and error fashion. In this case, even small improvements in care could represent a significant advance. Third, the interactions among genetic variants, medications and individual patients are sufficiently complex that *a priori* estimates of sensitivity and specificity may not accurately predict the outcome of a PGT trial. Only empirical data can determine the value of PGTs in clinical practice. Hence, we conclude that the potential for benefit from PGT is high, and will only increase as whole genome sequence information becomes widely incorporated into newer PGT panels and new genetic associations are discovered.

On the other side of the equation, the costs of repeated medication trials, prolonged illness, clinician time and unanticipated adverse drug events are also high, while the cost of genotyping, incurred only once, is relatively low, and likely to drop further. Therefore, while the potential for therapeutic gains may be modest in the first iterations of PGT, the downside risk imposed upon the patient is minimal, making the cost-benefit ratio strongly favorable. Therefore, carefully designed implementation studies constitute an essential and effective tool in addressing these concerns while playing an instrumental role in the validation process of genetic tests and their introduction into routine psychiatry practice. Implementation studies, as proposed here, will likely be a useful approach in deciding the value of a genetic test in real world clinical situations [162].

### Box 1: Pharmacogenetic association studies versus implementation studies

Genetic association studies have been used widely in psychiatric pharmacogenetics to determine statistical associations between genetic markers and a phenotype related to treatment outcome, typically treatment response, or a major side effect (for example, sexual dysfunction, Stevens-Johnson rash). These are done either as candidate gene studies where a specific hypothesis is tested or as GWAS in which associations sampled from across the whole genome are examined independent of a specific hypothesis. In association studies, patients are determined to be responder or non-responder in categorical or quantitative terms, an assessment that can be determined either prospectively or retrospectively. There is typically no comparator treatment used in association studies.

bibuIn comparison, genetic implementation studies are relatively rare in the psychiatry literature. These studies evaluate the clinical utility of a genetic test(s) versus a control treatment that does not utilize genetic information. Here, the association between the genetic marker to the phenotype is assumed, and the hypothesis tested is that the information provided by the genetic marker will allow for better drug selection than would occur in the absence of this information. Implementation studies have to be prospective in order to determine the effects of adding the information. The endpoints would be treatment response and/or side effect burden at the end of the trial.

## Abbreviations

ABCB1: gene for an ATP binding cassette transporter protein; AIM: antidepressant-induced mania; ANKK1: ankyrin repeat and kinase domain containing 1; BD: bipolar disorder; BDNF: brain-derived neurotrophic factor; bp: base pair; CBZ: carbamazepine; CREB: cyclic AMP response element; CYP450: family of proteins involved in drug metabolism; DRD2: gene for the D2 dopamine receptor; epS: extrapyramidal symptoms; FKBP5: gene for a co-chaperone protein for the glucocorticoid receptor; GRIA2: AMPA type glutamate receptor subunit; GRIK4: gene for the ionotropic kainate glutamate receptor; GSK3B: glycogen synthase kinase beta; GWAS: genomewide association study; HTR2A: gene for the serotonin 2A receptor; HTR2C: gene for the serotonin 2C receptor; MC4R: gene for the melanocortin 4 receptor; MDD: major depressive disorder; NTRK2: gene name for the TrkB receptor; ODZ4: gene for Odd Oz/Ten-m Homolog 4; PGT: pharmacogenetic guided treatment; SCZ: schizophrenia; SJS: Stevens-Johnson syndrome; SLC6A4: gene for the serotonin transporter; SNP: single nucleotide polymorphism; SSRI: selective serotonin reuptake inhibitor; TAU: treatment as usual; TrkB: tropomyosin related kinase B.

## Competing interests

The authors declare they have no competing interests.

## Authors’ contributions

NS conducted literature searches and wrote portions of the manuscript; MM was involved in conceptualization of the paper and wrote portions of the manuscript; SL conducted literature searches and helped draft the manuscript; JK helped in conceiving the paper and wrote portions of the manuscript. All authors read and approved the final manuscript.
